# Novel Use of an Orbital Atherectomy Device for In-Stent Restenosis: Lessons Learned

**DOI:** 10.1155/2016/5047981

**Published:** 2016-11-15

**Authors:** K. Shaikh, S. Kelly, M. Gedela, V. Kumar, A. Stys, T. Stys

**Affiliations:** USD Department of Medicine, Division of Cardiology, Sioux Falls, SD, USA

## Abstract

We present a case of a 67-year-old man with stage III chronic kidney disease, uncontrolled diabetes mellitus, coronary artery disease, and high surgical risk who presented with two episodes of acute coronary syndrome attributed to in-stent restenosis (ISR) associated with heavily calcified lesions. In this case, we were able to improve luminal patency with orbital atherectomy system (OAS); however, withdrawal of the device resulted in a device/stent interaction, causing failure of the device. Given limitations in current evidence and therapies, managing ISR can be a technical and cognitive challenge. Balloon expansion of the affected region often provides unsatisfactory results, possibly related to significant calcium burden. OAS could be an efficacious way of reestablishing luminal patency in ISR lesions, as these lesions are often heavily calcified.

## 1. Introduction

ISR is a pathological development of neointimal hyperplasia resulting in the progressive loss of luminal area. Recent studies have suggested that early phase neointimal hypertrophy is in part due to calcification. These lesions are more robust and may contribute to stent under expansion if treated with balloon angioplasty alone [1, 2]. The management of ISR is complex, with a few treatment modalities recognized other than the balloon angioplasty and further stent placement. Unfortunately, implementation of these techniques can be ineffective in the presence of heavily calcified lesions. Calcified lesions are challenging as they can be associated with increased rates of stent restenosis due to being under expansion and poor apposition, which predispose to luminal loss and worse clinical outcome [1, 3]. OAS was recently introduced to the US market for the management of heavily calcified coronary lesions. Though recently approved, its five years of clinical use has been associated with a good safety profile and reasonable patency rates [4]. Previous rotational atherectomy devices, when used with adjuvant balloon angioplasty, have demonstrated safety and efficacy for the management of ISR occurring in femoral arteries [5]. However, the use of orbital or rotational atherectomy devices in the management of ISR remains off-label.

## 2. Case Presentation

A 67-year-old male with coronary artery disease, New York Heart Association Class III diastolic heart failure, paraplegia, diabetes mellitus, and stage III kidney disease presented with a high-risk, non-ST segment elevation myocardial infarction (NSTEMI) and worsening renal failure. The patient had presented six months earlier with an NSTEMI attributed to in-stent restenosis. Angiography at that time revealed calcified lesions, in addition to a 90% in-stent restenosis within the left anterior descending artery (LAD). These lesions were treated with primary balloon angioplasty and the placement of two drug-eluting stents within the culprit region of the LAD. The patient was subsequently discharged on dual antiplatelet therapy.

**Figure 1:**
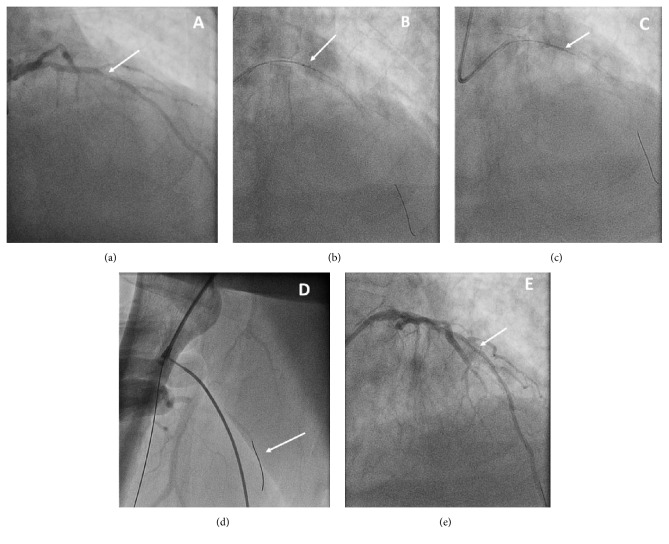
(a) RAO caudal projection, identifying 90% in-stent restenosis of LAD (white arrow). (b) RAO cranial projection showing orbital atherectomy system engaged in stent (white arrow). (c) Radiopaque distortion of device head showing unraveling of driveline coils (white arrow). (d) Viper wire embolized in small distal right profunda femoris artery (white arrow). (e) RAO cranial projection, final result, showing widened luminal area of in-stent restenosis (white arrow).

His most recent presentation was characterized by features of NSTEMI including typical cardiac chest pain, elevated troponins, and worsening renal function. The patient initially received goal-directed medical therapy and was monitored for a few days until his renal function recovered enough to permit iodinated contrast administration. Subsequent cardiac catheterization revealed disease in the right coronary artery of 65% distal and 90% bifurcating lesions, 90% small circumflex, and 90% in-stent restenosis of the mid LAD (Figure 1(a)). Cardiothoracic surgery was consulted for revascularization with coronary artery bypass. Due to multiple comorbidities, the patient was deemed a poor surgical candidate. The decision was thus made to treat the heavily calcified neointimal hyperplastic segment using the Diamond Back® orbital atherectomy system with adjuvant balloon angioplasty and stent placement.

**Figure 2:**
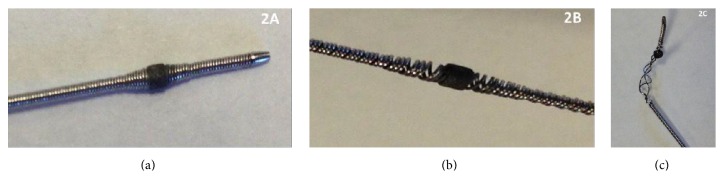
(a) Picture demonstrating normal orbital atherectomy device composed of coiled wires and sanding disc coiling of supportive wires provide radial strength and flexibility. (b) Picture showing an unused device with the creation of potential spaces caused by mild traction of the device. This space is the likely area on which the stent strut was caught during retraction of the device, thereby facilitating the unwinding process. (c) Picture of the unwound device showing three supportive struts that are no longer coiled.

The percutaneous coronary intervention was performed using a 6-French guide catheter and the Diamond Back orbital atherectomy device (Figure 2(a)). The lesion was crossed using a hydrophilic workhorse wire and exchanged for the ViperWire® using a Corsair microcatheter exchange technique. The orbital atherectomy cutting head was advanced proximal to the area of in-stent restenosis contained within the confines of the stent (Figure 1(b)). The device was then activated and the head was successfully advanced across the stenotic segment on several passes. However, during retraction of the device from the proximal stented region, a radiopaque distortion of the device head was noted (Figure 1(c)) and the procedure was aborted (Figure 2(b)).

After withdrawal of the device from the catheter system, it was observed that the orbital head was enveloped with wires that had been severed from the braided driveline. The procedure was further complicated by embolization into the femoral artery of the distal segment of the ViperWire (Figure 1(d)) that occurred during the wire exchange. Nonetheless, it did show widening of the lumen (Figure 1(e)).

## 3. Discussion

Over the past several years, innovations in interventional techniques and devices have afforded physicians the ability to address complex coronary interventions with improvements in clinical outcomes. Despite these advancements, highly calcified coronary lesions still remain a complex clinical entity that present challenges for current coronary devices and techniques. In addition to providing technical challenges, calcified lesions are associated with poorer clinical outcomes including increased frequency of myocardial infarction and in-stent restenosis, which are likely related to issues of poor stent expansion and wall approximation. Indeed, stent under expansion, asymmetric expansion, and stent malposition are frequently found in the intravascular ultrasound (IVUS) evaluations of heavily calcified plaques [3]. Indicators of angiographic success and presumptive benefit such as residual stenosis are worse in heavily calcified lesions compared to noncalcified plaques [1, 3, 6]. There are several cases reported that demonstrate early phase neointimal calcification in ISR lesions, which are more common in patient with diabetes mellitus and chronic kidney disease [1, 2]. OAS was recently approved by the FDA for the treatment of severely calcified de novo coronary lesions and it has exhibited encouraging outcomes and safety data [4]. This device uses a diamond-coated abrasive head that when rotating exerts a centrifugal force capable of pulverizing calcified deposits. The head is guided over a rigid proprietary wire and has the perceived advantage of cutting in either a forward or reverse direction. Use of OAS and stent placement in challenging calcified lesions were associated with lower major cardiovascular event (MACE) rates in the ORBIT I trial (12.1% at 6 months, 15.2% at 2 years, 18.2% at 3 years, and 21.2% at 5 years) as opposed to the Rotaxus trial MACE rate which was 24.9% at 9 months in treated complex calcified lesions [4].

Use of OAS in the management of in-stent restenosis is off-label in the United States. To our knowledge, there are no studies or case reports documenting the use of OAS in ISR, although there is some data demonstrating both the safety and efficacy of a rotational atherectomy device used in infrainguinal ISR lesions [5].

Use of the Jetstream® rotational atherectomy device in the infrainguinal vessels demonstrated favorable patency rates and was not associated with adverse outcomes, such as stent perforation or device/stent interactions [5]. To date, no randomized controlled trial has compared the OA versus RA. Although understanding the differential impact of OA and RA on patient outcomes awaits results of such a trial, early insights from plaque imaging following ablation suggest differences between the two techniques in their effects on calcified lesions. In a study of 20 consecutive patients with OCT imaging before and after OA (*n* = 10) or RA (*n* = 10), OA was associated with deeper dissections, particularly in plaques composed of comparatively more lipid and less calcium. In this small study, there was also a signal toward improved stent expansion following OA; in comparison with RA-treated patients, OA-treated patients had a significantly lower incidence of stent strut malapposition (4 versus 8%, *p* = 0.038). Whether this distinction will lead to long-term reductions in target lesion failure remains to be confirmed by a prospective, randomized controlled trial. Multicenter Prospective Study to Evaluate Outcomes of the Moderate to Severely Calcified Coronary Lesions (MACE) is needed, which will make available observational data about the real-world application, comparative safety, efficacy, and costs of different treatment strategies for de novo calcified lesions, including OA and RA [7, 8].

The patient in our case represented a difficult situation because he had multiple medical ailments prohibiting surgical revascularization and had also endured a previous NSTEMI attributed to in-stent restenosis in a calcified artery. As he was a high surgical risk and rotational atherectomy has been proven efficacious in the treatment of infrainguinal ISR, OAS seemed to be a viable option. OAS did improve luminal patency; however, as noted, the procedure was prematurely aborted due to device failure attributed to stent/device interaction. The embolization of the distal end of the wire was likely related to a fracture of the wire occurring from initial device failure. It has been claimed that the device failure can be attributed to the orbital head skirting behind a stent strut (Figure 2(c)).

This could suggest that the workhorse wire initially entered behind a strut with initial cannulation of the vessel. We suggest that using an IVUS with the workhorse wire would allow for visualization of the wire's course and prevent inadvertent deflection of the wire behind the strut. Secondly, IVUS is considerably more accurate than angiography for coronary artery calcification location and permits accurate determination of the calcification burden and vascular dimension [2, 7, 8].
